# The complete chloroplast genome sequences of five pinnate-leaved *Primula* species and phylogenetic analyses

**DOI:** 10.1038/s41598-020-77661-3

**Published:** 2020-11-27

**Authors:** Wenbin Xu, Boshun Xia, Xinwei Li

**Affiliations:** Wuhan Botanical Garden, Chinese Academy of Sciences, Wuhan, 430074 China

**Keywords:** Ecology, Evolution, Plant sciences

## Abstract

The six pinnate-leaved species are a very particular group in the genus *Primula.* In the present paper, we sequenced, assembled and annotated the chloroplast genomes of five of them (*P. cicutarrifolia*, *P*. *hubeiensis*, *P. jiugongshanensis*, *P. merrilliana*, *P*. *ranunculoides*). The five chloroplast genomes ranged from ~ 150 to 152 kb, containing 113 genes (four ribosomal RNA genes, 29 tRNA genes and 80 protein-coding genes). The six pinnate-leaved species exhibited synteny of gene order and possessed similar IR boundary regions in chloroplast genomes. The gene *accD* was pseudogenized in *P*. *filchnerae*. In the chloroplast genomes of the six pinnate-leaved *Primula* species, SSRs, repeating sequences and divergence hotspots were identified; *ycf1* and *trnH*-*psbA* were the most variable markers among CDSs and noncoding sequences, respectively. Phylogenetic analyses showed that the six *Primula* species were separated into two distant clades: one was formed by *P*. *filchnerae* and *P*. *sinensis* and the other clade was consisting of two subclades, one formed by *P*. *hubeiensis* and *P*. *ranunculoides*, the other by *P. merrilliana*, *P. cicutarrifolia* and *P*. *jiugongshanensis*. *P*. *hubeiensis* was closely related with *P*. *ranunculoides* and therefore it should be placed into Sect. *Ranunculoides*. *P. cicutarrifolia* did not group first with *P*. *ranunculoides* but with *P. merrilliana*, although the former two were once united in one species, our results supported the separation of *P*. *ranunculoides* from *P. cicutarrifolia* as one distinct species.

## Introduction

*Primula* L. consists of about 430 species (seven subgenera, 38 sections) in the world^1^, and there are 300 species (24 sections) in China^2^. Altogether six species have leaves pinnately compound or pinnately lobed to the midvein: *P. cicutarrifolia*, *P*. *filchnerae*, *P*. *hubeiensis*, *P*. *jiugongshanensis*, *P*. *merrilliana* and *P*. *ranunculoides*^3–5^. These species are all endangered^3,4,6–9^. The ITS (internal transcribed spacer) phylogeny trees showed that *P*. *filchnerae* should be placed in Sect. *Auganthus*, and *P*. *cicutarrifolia, P*. *merrilliana* and *P*. *jiugongshanensis* belonged in Sect. *Ranunculoide*s^4,10,11^. *P*. *hubeiensis* might attribute to Sect. *Auganthus*^5^. Chloroplast fragments *matK*, *rps16*, and *trnL*-*F* data also supported *P*. *filchnerae* to be included in Sect. *Auganthus*^12^*.* Based on the ITS phylogeny that revealed *P. fichnerae*, *P*. *cicutarrifolia* and *P*. *merrilliana* not to cluster into a monophyly Hao et al. (2002)^10^ suggested that the character of pinnately lobed or divided leaves had evolved in parallel. However, the phylogenetic relationships among the six pinnate-leaved species were not explored yet.

The chloroplast (cp) genomes possess conserved structure including two copies of an inverted repeat regions (IRs) linking large and small single-copy regions (LSC and SSC)^13^. Due to moderate substitution rate^14^, molecular markers derived from cp genomes are widely used in plant population genetics, molecular phylogenetics, evolutionary biology and species identification. The complete cp genomes could provide higher phylogenetic resolution than ITS or selected chloroplast DNA data^15–17^, chloroplast genomic data provided strong support for resolution of controversial phylogenetic relationships^18,19^. A few cp genomes were reported for *Primula* species including *P*. *filchnerae*^20,21^. In the present study, we will release five complete cp genomes of pinnate-leaved *Primula* species, comparing their genome contents and structure, exploring SSRs and repeats, identifying variable regions, in order to facilitate conservation and systematics of the genus *Primula* .

## Materials and methods

### Genome sequencing, assembly and annotation

We collected six species in China: *P*. *cicutarrifolia* in Hangzhou, Zhejiang, *P*. *merrilliana* in Mt. Huang, Anhui, *P*. *hubeiensis*, *P*. *jiugongshanensis* and *P*. *ranunculoides* in Tongshan, Hubei, and *P*. *filchnerae* in Zhuxi, Hubei*.*

DNA was isolated from fresh leaves using CTAB method^22^. Paired-end libraries with about 350 bp DNA insertion were prepared using Illumina TruSeq Library preparation kits (Illumina, San Diego, CA, USA) according to the manufacturer’s protocol. The libraries were sequenced on the Illumina Hiseq 2500 platform (Illumina Inc.), generating raw data of 150 bp paired-end reads.

The raw data were subjected to quality control using NGS QC Toolkit^23^ (cut-off value for PHRED quality score = 30), then the filtered data were imported into CLC Genomics Workbench v. 11.0.1 (https://www.qiagenbioinformatics.com) to generate contigs with the word value of 60. The relative order and orientation of the contigs of the cp genomes of five species (*P*. *cicutarrifolia*, *P*. *merrilliana*, *P*. *hubeiensis*, *P*. *jiugongshanensis* and *P*. *ranunculoides*) were determined by BLAST search against the cp genome of *P. sinensis* (NC_030609). The hit contigs were then concatenated into complete sequences with minimum overlap of 31 bp in Geneious 9 (Biomatters, Auckland, New Zealand); gaps between contigs were closed by comparison with the contigs produced by IOGA^24^ with *P. sinensis* (NC_030609) as the reference. The filtered data were mapped back onto the newly assembled cp genomes to confirm no assembly errors by the Geneious plugin in Geneious 9 (Biomatters, Auckland, New Zealand). The cp genome of *P*. *filchnerae*^21^ was downloaded from NCBI because we collected the sample of *P*. *filchnerae* from same place as Sun et al.^21^.

The ITS (Internal Transcribed Spacer) sequences of the six species were generated from the consensus of the reads of the quality controlled data mapped onto that of *P*. *sinensis* (JF978052) by the Geneious plugin in Geneious 9 (Biomatters, Auckland, New Zealand).

The cp genomes in this study were annotated using GeSeq^25^, choosing the MPI-MP chloroplast references as the references. The annotations were modified manually by comparing with Primulaceae cp genomes available in the Genbank. The cp genome maps were drawn using OGDRAW^26^. The sequences of the cp genomes were visualized in Geneious 9 (Biomatters, Auckland, New Zealand). The synteny of the cp genomes of six *Primula* species with pinnatisect leaves was estimated with MAUVE 20150226^27^.

All alignment was done with MAFFT^28^ for further analyses.

### Repeat element analysis

Simple sequence repeats (SSR) were detected for the cp genomes using MISA^29^. The minimum numbers for the SSR motifs were 10, 5, 4, 3, 3 and 3 for mono-, di-, tri-, tetra-, penta-, and hexa- nucleotide repeats, respectively. REPuter^30^ was used to identify the repeating sequences (forward, reverse, complement and palindrome) with three for Hamming distance, 30 for Minimal Repeat Size.

### Sequence divergence analysis

We calculated the nucleotide variability (Pi) values of the protein coding sequences, introns and intergenic spacers of the cp genomes of the six species with pinnatisect leaves and 16 other *Primula* species available in Genbank (Table S1) using DnaSP 6.12^31^.

### Phylogenetic analysis

We constructed phylogenetic trees by Maximum likelihood (ML) and neighbor-joining (NJ) methods. *Androsace paxiana* and *Lysimachia congestiflora* were treated as outgroups. The ML phylogenetic trees (1000 bootstrap replicates) were inferred with RAxML 8.2.10^32^ based on whole cp genomes (Tables S1), *ycf1*, and concatenation of ITS, *matK* and *rbcL* (Tables S2), respectively. *ycf1* was extracted from the cp genomes (Tables S1)*,* because the gene *ycf1* did not exist in *P*. *tsiangii*, we used its homologous part in the cp genome. NJ analysis of 71 *Primula* species including the six species with pinnatisect leaves based on concatenation of ITS, *matK* and *rbcL* (Tables S2) was carried out using MEGA-X^33^ with 1000 bootstrap replicates. ITS of the six pinnate-leaved *Primula* species was newly sequenced in this study; *matK* and *rbcL* were derived from the chloroplast sequences in this study and from the cp genome of *P*. *filchnerae*^21^. The accessions of the cp genomes and DNA fragments were listed in Tables S1, S2, respectively.

## Results

### Basic characters of the six chloroplast genomes

The cp genomes of *P*. *cicutarrifolia*, *P*. *hubeiensis*, *P*. *jiugongshanensis*, *P*. *merrilliana* and *P*. *ranunculoides* (GenBank accessions: MT268974, MT268976, MT937162, MT268977, MT268978) were reported for the first time here, and that of *P*. *filchnerae* was downloaded from NCBI (MK888698^21^).

**Figure 1:**
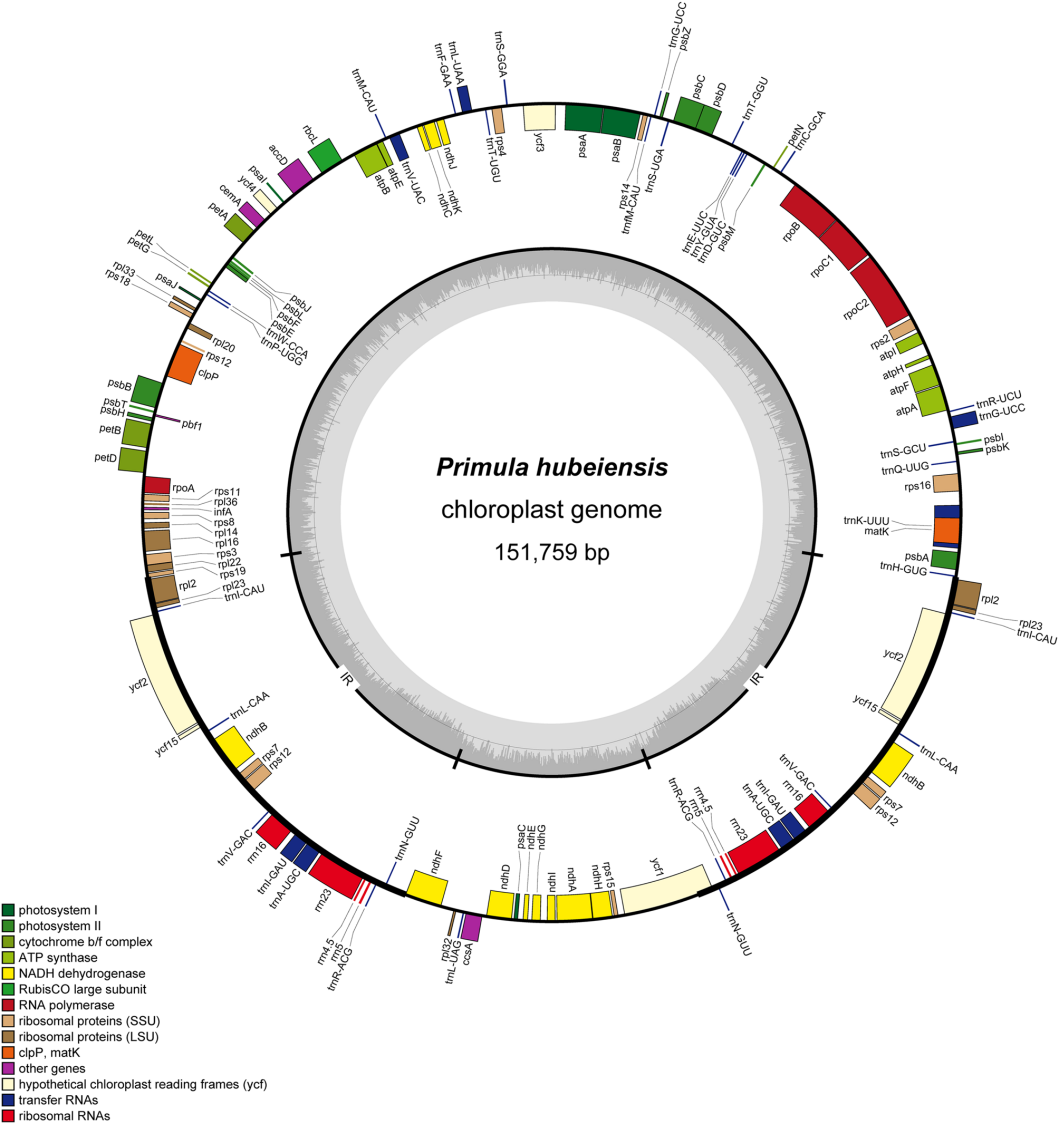
Physical map of the *P*. *hubeiensis* chloroplast genome.

The sequencing coverage of our five newly assembled cp genomes was from 923 to 6237 (Figure S1). The six cp genomes possessed typical quadripartite structure: IRa, IRb, LSC and SSC (Table 1), and they exhibited the same gene order, no gene rearrangement or inversion occurred (Figure S2). The physical map of the cp genome of *P*. *hubeiensis* was shown in Fig. 1. The GC content was ~ 37%. The newly sequenced genomes ranged from 150,187 bp to 151,972 bp, harboring 113 genes: four ribosomal RNA genes, 29 tRNA genes and 80 protein-coding genes, and among them 14 genes was duplicated in IRa and IRb (Table 1). Due to presence of multiple stop codons, the gene *infA* was pseudogenized in the five newly sequenced species. The open reading frame (ORF) in *accD* of *P*. *filchnerae* (MK888698) was truncated to be only 1305 bp compared with 1455 or 1464 bp ORF of other five species. Lee et al*.*^34^ identified five conserved amino acid sequence motifs in *accD* gene. Conserved amino acid sequence motifs IV and V were absent in *accD* of *P*. *filchnerae*. Therefore, *accD* was nonfunctional in *P*. *filchnerae*.

**Table 1:** Basic characteristics of cp genomes of the six *Primula* species (Pc: *P*. *cicutarrifolia*; Pf: *P*. *filchnerae*; Ph: *P*. *hubeiensis*; Pj: *P*. *jiugongshanensis*; Pm: *P*. *merrilliana*; Pr: *P*. *ranunculoides*).

Species	Pc	Pf	Ph	Pj	Pm	Pr
Total length	151,972 bp	151,547 bp	151,759 bp	151,696 bp	151,843 bp	150,187 bp
GC%	36.8%	37.2%	36.8%	36.8%	36.8%	36.8%
LSC	83,945 bp	82,662 bp	83,523 bp	83,797 bp	83,847 bp	82,031 bp
SSC	17,839 bp	17,749 bp	17,632 bp	17,521 bp	17,554 bp	17,572 bp
IR	25,094 bp	25,568 bp	25,302 bp	25,189 bp	25,221 bp	25,292 bp
Total genes	113	112	113	113	113	113
Protein genes	80	79	80	80	80	80
rRNA genes	4	4	4	4	4	4
tRNA genes	29	29	29	29	29	29

### SSRs and repeats

Five categories of SSRs were identified for the six species (Table 2). The least number of SSRs was 41 for *P*. *ranunculoides* and the most 59 for *P*. *merrilliana*. Three types of SSRs were detected for *P*. *filchnerae*, and in the rest species four types could be found. While mono-, di- and tetra-nucleotide repeats existed across all the six species, tri- and penta-inucleotide repeats resided in three and two species respectively. Mono- and dinucleotide repeats accounted for the vast majority of SSRs (65.1% for *P*. *cicutariifolia*, 87.5% for *P*. *filchnerae*, 69.0% for *P*. *hubeiensis*, 62.8% for *P*. *jiugongshanensis*, 72.9% for *P*. *merrilliana*, 73.2% for *P*. *ranunculoides*). Most or all mono- repeats were A/T repeats including 10 to 16 nucleotides. The number of repeat units ranged from five to eight for dinucleotide repeats. The tri- and penta-nucleotide SSRs consisted of four motifs, and tetra-nucleotide SSRs of four to five repeat units.

**Table 2:** Types and numbers of SSRs in the cp genomes of six *Primula* species, the numbers in the bracket indicating total number of SSRs (Pc: *P*. *cicutarrifolia*; Pf: *P*. *filchnerae*; Ph: *P*. *hubeiensis*; Pj: *P*. *jiugongshanensis*; Pm: *P*. *merrilliana*; Pr: *P*. *ranunculoides*).

Type	Repeat unit	Pc (43)	Pf (56)	Ph (42)	Pj (43)	Pm (59)	Pr (41)
Mono	A/T	27	49	29	27	42	29
	C/G	1	–	–	–	1	1
Di	AT/AT	8	5	9	9	8	8
Tri	AAG/CTT	2	–	–	2	2	–
Tetra	AAAT/ATTT	2	2	2	2	4	1
	AACG/CGTT	2	–	–	2	2	–
	AATT/AATT	1	–	–	–	–	–
	AGAT/ATCT	–	–	–	1	–	–
	AAAG/CTTT	–	–	–	–	–	1
Penta	AATGT/ACATT	–	–	1	–	–	1
	AAAAT/ATTTT	–	–	1	–	–	–

**Figure 2:**
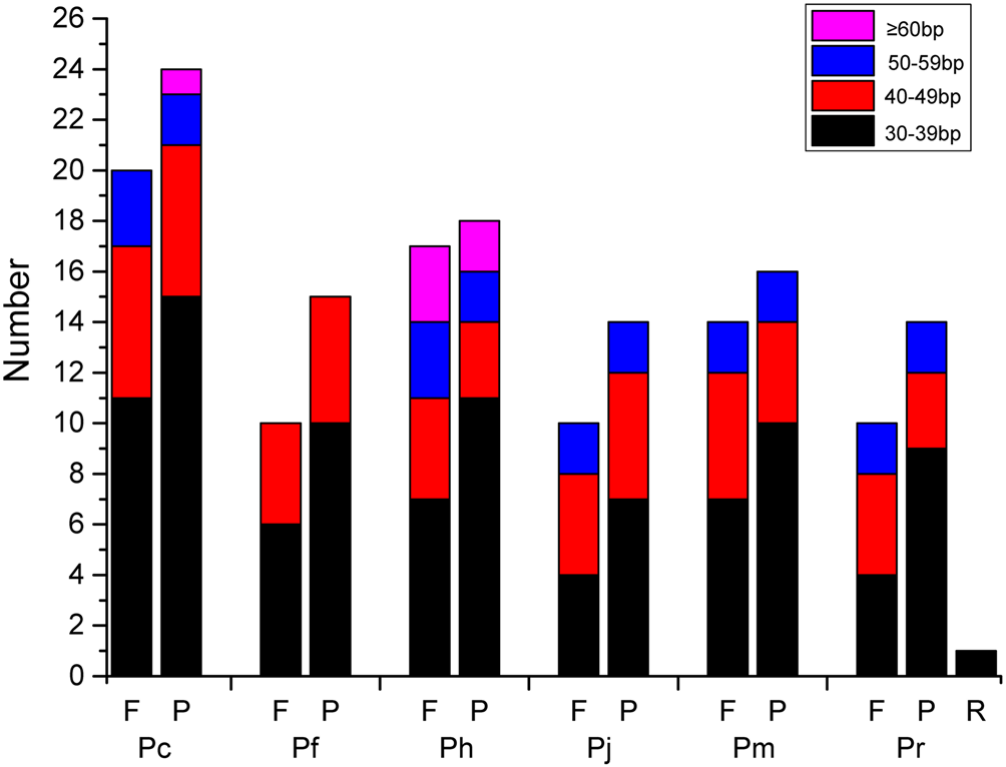
Types and numbers of repeat pairs in the cp genomes of six *Primula* species (Pc: *P*. *cicutarrifolia*; Pf: *P*. *filchnerae*; Ph: *P*. *hubeiensis*; Pj: *P*. *jiugongshanensis*; Pm: *P*. *merrilliana*; Pr: *P*. *ranunculoides*).

Except the largest repeat for each genome (i.e. IRs), a total of 183 repeat pairs (three types: forward (F), reverse (R), and palindromic repeats (P)) were detected in the six genomes (Fig. 2), which ranged from 30 to 137 bp in length. Palindromic repeats were the most common, accounting for 55.2% (101 of 183), followed by forward repeats (44.3%, 81 of 183). No complement repeats were identified in all species and one pair of reverse repeats existed specifically in *P*. *ranunculoides*. In the six species, 96.7% (177 of 183 repeat pairs) repeats were 30–59 bp in length, consistent with the length reported in other *Primula* species^20^. The longest repeat (137 bp) was found in *P*. *cicutariifolia*, and this species contained the most repeats (44 pairs), while *P*. *jiugongshanensis* had the least (24 pairs).

### IR/SC boundary

**Figure 3:**
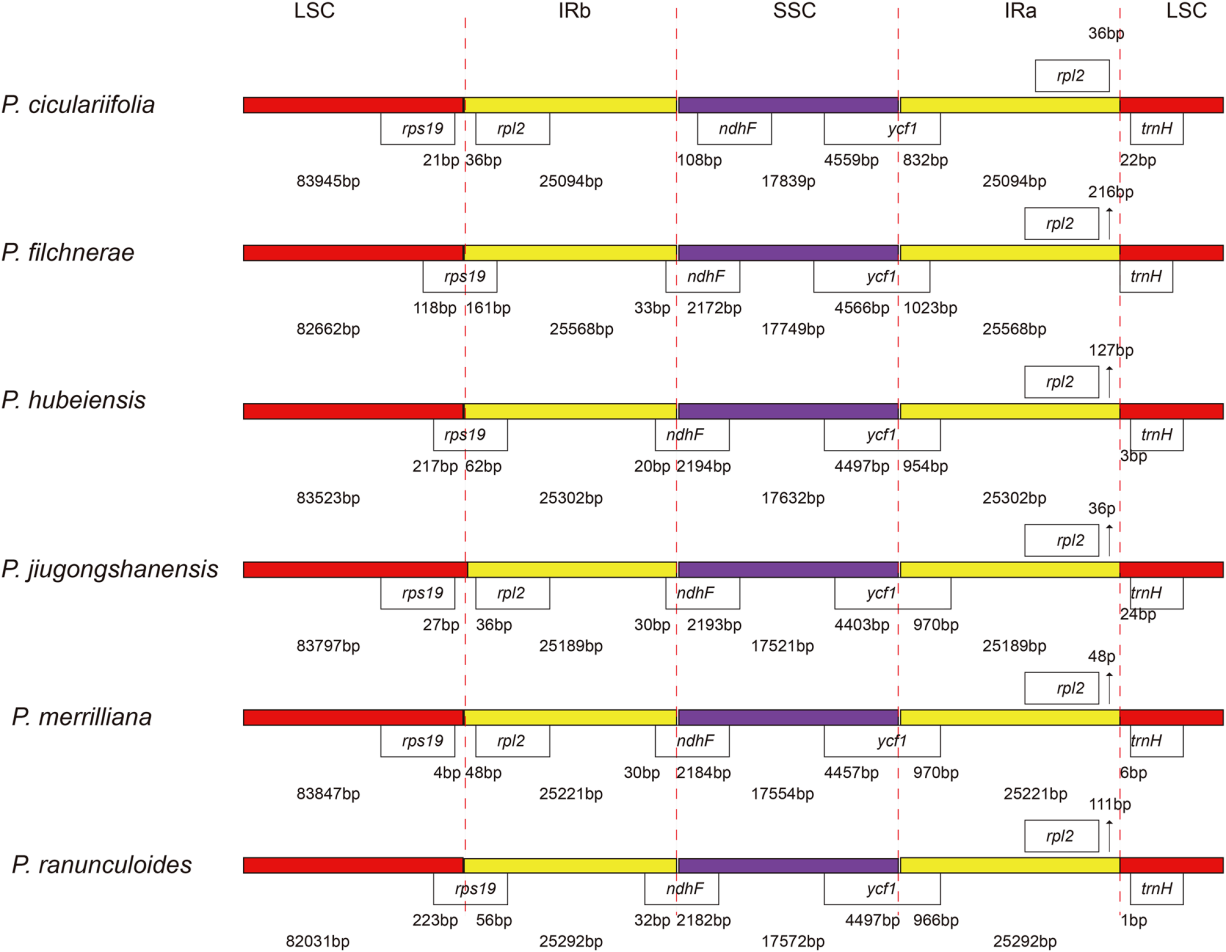
LSC/IR, and SSC/IR border regions of the six *Primula* cp genomes.

The IR/SC boundary regions of the six *Primula* cp genomes were compared, and the IR/SC junction regions showed slight differences in the length of organization genes flanking the junctions or the distance between the junctions and the organization genes (Fig. 3). The genes spanning or flanking the junction of LSC/IRb, IRb/SSC, SSC/IRa and IRa/LSC were *rps19*/*rpl2*, *ndhF*, *ycf1*, *rpl2*/t*rnH*, respectively. IR expansion and contraction was observed. *P*. *cicutarrifolia* had the smallest size of IR but largest size of both LSC and SSC; though largest size of IR was detected in *P*. *filchnerae*, the LSC or SSC was not the smallest in this species. The gene *trnH* was located in LSC, 0–24 bp away from the IRa/LSC border. The largest extensions of *ycf1* into both SSC and IRa occurred in *P*. *filchnerae* (4566 bp and 1023 bp, respectively) and *ycf1* of *P. filchnerae* were the longest among the six species. The gene *ndhF* was utterly situated in SSC and 108 bp distant from the IRb/SSC junction in *P*. *cicutarrifolia*; in the rest five species the fragment size of *ndhF* in SSC was largest in *P*. *hubeiensis* (2194 bp). In *P*. *cicutarrifolia*, *P*. *jiugongshanensis* and *P. merrilliana*, *rps19* and *rpl2* were located in the upstream and downstream of the LSC/IRb junction, respectively; *rps19* ran across the LSC/IRb junction in *P*. *filchnerae*, *P*. *hubeiensis*, *P*. *ranunculoides* with 161, 62, 56 bp extension in IRb, respectively.

### Divergent hotspots in the *Primula* chloroplast genome

As indicated by the value of Pi, the nucleotide variability of the 22 *Primula* species (Table S1) was evaluated by DnaSP 6.12^31^ using noncoding sequences (intron and intergenic spacer) or protein coding sequences (CDS) at least 200 bp long. The variation level of DNA polymorphorism was 0.00444–0.11369 for noncoding sequences or 0.00094–0.05036 for CDSs. For the CDSs, the highest Pi value were detected for *ycf1* (0.05036), followed by *matK* (0.04878), *rpl22* (0.04364), *ndhF* (0.03975), *rps8* (0.03658), *ndhD* (0.03455), *ccsA* (0.03292), *rpl33* (0.0303), *rps15* (0.03022), and *rpoC2* (0.02954). These markers had higher Pi than *rbcL* (0.02149). Obviously, the gene *ycf1* exhibited the greatest diversity and harbored the most abundant variation. The ten most divergent regions among noncoding regons included *trnH* (GUG)-*psbA* (0.11369), *trnW* (CCA)-*trnP* (UGG) (0.09463), *rpl32*-*trnL* (UAG) (0.09337), *ndhC*-*trnV* (UAC) (0.09148), *ccsA*-*ndhD* (0.08745), *ndhG*-*ndhI* (0.08363), *trnK* (UUU)-*rps16* (0.08334), *trnM* (CAU)-*atpE* (0.08273), *trnS* (GGA)-*rps4* (0.08028), and *trnC* (GCA)-*petN* (0.07971). No intron ranked among the top ten variable noncoding regions.

### Phylogenetic analysis

**Figure 4:**
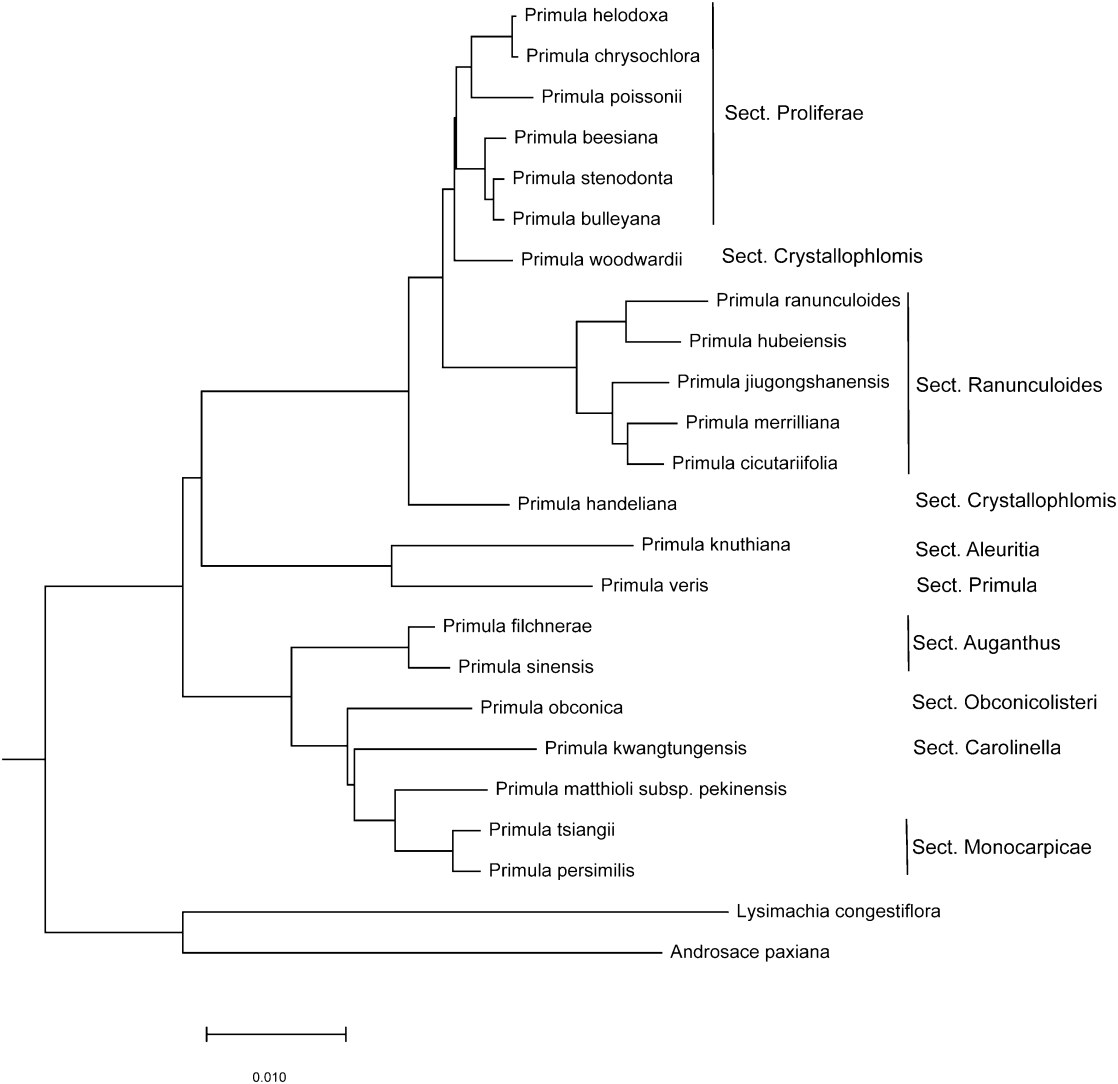
ML phylogenetic tree of *Primula* species based on cp genomes. Bootstrap support at nodes are all 100%.

The ML tree of 22 *Primula* species was constructed with RAxML^32^ (Fig. 4), based on the whole cp genomes. The six pinnate-leaved *Primula* species did not form a monophyly, but separated into two distant clades. *P*. *filchnerae* grouped with *P*. *sinensis*, the other five species clustered together and constituted the clade Sect. *Ranunculoides* with 100% bootstrap. In the ML tree, Sect. *Proliferae* exhibited monophyly, while species of Sect. *Crystallophlomis* separated into different clades.

The topology of the ML tree based on *ycf1* (Figure S3) was consistent with that based on whole cp genomes (Fig. 4), except that the clade formed by *P*. *veris* and *P*. *knuthiana* were sister to the clade consisting of Sects. *Auganthus, Obconicolisteri, Carolinella* and *Monocarpicae* instead of being sister to the clade of Sects. *Proliferae, Ranunculoides* and *Crystallophlomis*.

**Figure 5:**
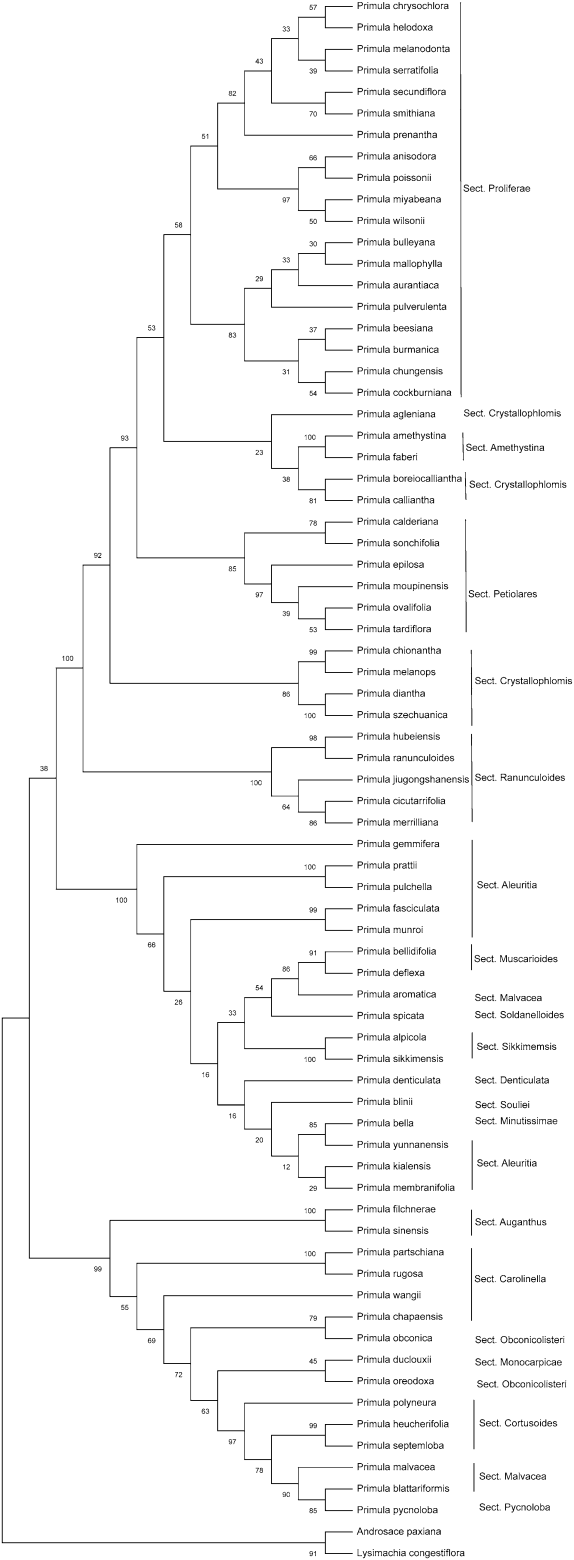
NJ bootstrap consensus tree of *Primula* based on concatenation of ITS, *matK* and *rbcL*.

We also constructed both ML and NJ tree of 71 *Primula* species based on the concatenation of three common barcoding markers (ITS, *matK* and *rbcL*). Only the results of NJ analysis (Fig. 5) showed consistency with those of Yan et al.^12^, Liu et al.^35^, and ML analysis based on whole cp genomes (Fig. 4). The six pinnate-leaved *Primula* species were separated into two distantly related groups. The clade consisting of *P*. *filchnerae* and *P. sinensis* (Sect. *Auganthus*) was sister to the clade formed by Sects. *Carolinella*, *Obconicolisteri*, *Monocarpicae*, *Cortusoides, Malvacea*, *Pycnoloba.* The five pinnatisect-leaved species *P. cicutarrifolia*, *P. hubeiensis*, *P*. *jiugonshanensis*, *P. merrilliana and P. ranunculoides* (Sect. *Ranunculoides*) comprised a 100% supported clade, which was sister to the group containing Sects. *Crystallophlomis*, *Petiolares*, *Proliferae*, *Amethystina*. Sect. *Carolinella* and Sect. *Crystallophlomis,* and Sect. *Malvacea* were polyphyletic.

## Discussion

The six cp genomes of pinnate-leaved species were ~ 150–152 kb with similar GC content. The gene content and organization were similar and a high degree of synteny in gene order was observed across all the genomes. The gene *accD* was normal in five species but perhaps pseudogenized duo to lack of two conserved amino acid sequence motifs in *P*. *filchnerae.* In *P*. *sinensis*, this gene was pseudogenized and another copy of *accD* were detected in the nucleus^36^. Whether *accD* was functionally transferred to the nucleus in *P*. *filchnerae* needs further confirmation*.* Interestingly, *P*. *filchnerae* and *P*. *sinensis* always grouped together on the phylogenetic trees (Figs. 4, 5).

In the six *Primula* species, the IR/SC boundary regions exhibited similar feature, with slight differences observed in the length of organization genes flanking the junctions or the distance between the junctions and the organization genes (Fig. 3), and the situation is similar to ten other *Primula* species^20^, which indicates the structural conservation of *Primula*. Expansion of IR regions may cause size increase in chloroplast genomes^37^, however, it seems that the size of whole cp genomes did not always increase with expansion of IR in *Primula*. For example, among the six pinnate-leaved species, *P*. *cicutarrifolia* possessed the smallest IR (25,094 bp) but the largest whole genome size (151,972 bp); in *P*. *filchnerae*, the IR was the longest (25,568 bp), and the whole genome size (151,547 bp ) was only bigger than *P*. *ranunculoides* (150,187 bp). In *P*. *kwangtungensis*^20^, both IR (25,855 bp) and the whole genome size (153,757 bp) exceeded all other species (Table S1) including the six pinnate-leaved species.

Except the IRs, 183 pairs of repeats were detected in the six cp genomes, only one which were longer than 70 bp (137 bp), which is similar in ten other *Primula* species, most of repeats ranged in size from 14 to 62 bp and all (except one pair of 111 bp repeat) were not large repeats (> 100 bp)^20^. No rearrangement was found in our six species, the reason may be lack of large complex repeating sequences (> 100 bp) just as suggested by Ren et al.^20^. The SSR marker analyses have been proven to be powerful to assess the genetic diversity and population structure of *P*. *cicutarrifolia*, *P*. *merrilliana and P*. *sikkimensis*^7,38,39^. The usefulness of the SSRs located in the six chloroplast genomes may be tried in future studies on population genetics of *Primula* species.

Using the six pinnate-leaved species cp genomes and 16 other *Primula* cp genomes available in NCBI, the divergence hotspots were identified among CDSs and noncoding regions. The nucleotide diversity (Pi) of *ycf1* and *matK* reached 0.05036 and 0.04878, respectively, much higher than *rbcL* (0.02149), which was a common barcode for species identification. The gene *ycf1* was considered to be the most promising barcode to identify plant species^40^. Two chloroplast genes, *ycf1* and *psbM*-*psbD*, had much better discriminatory power (both 87.5%) than did other chloroplast barcodes for identifying *Fritillaria* species^41^. The ML species tree based on *ycf1* (Figure S3) showed similar topology to that based on whole cp genome. Except *matk* CDS, other hotspots regions identified here were not tested for species identification or phylogeny reconstruction^42,43^. Among the noncoding sequences, *trnH* (GUG) -*psbA* was the most variable one, which showed better discriminatory power than *matK* and *rbcL*^43^. These highly variable regions have the potential to be used for *Primula* species discrimination or phylogeny investigation in future study.

Both ML and NJ phylogenetic analyses revealed that the six pinnate-leaved *Primula* species did not form a monophyletic group, probably due to parallel evolution of pinnately lobed or divided leaves^10^. In the ML and NJ trees, the phylogenetic placement of the clade consisting of *P*. *filchnerae* and *P*. *sinensis* was near to Sect. *Carolinella* and Sect. *Obconicolisteri*, which is similar to the results of Yan et al.^12^. Liu et al.^35^ proposed Subgen. *Auganthus* (Sect. *Auganthus*, *Bullatae*, *Cortusoides*, *Dryadifolia*, *Malvacea*, *Monocarpicae*, *Obconicolisteri*, *Pycnoloba*) to include Subgen. *Carolinella* (Sects. *Carolinella*) and exclude *P*. *aromatica*, *P*. *filchnerae* and *P*. *sinensis* thus were in the basal clade of Subgen. *Auganthus.* The close relatedness of *P*. *filchnerae* and *P*. *sinensis* was also indicated by the pseudogenization of the gene *accD*. And our study showed that Sect. *Ranunculoides* (*P*. *cicutarrifolia*, *P*. *hubeiensis*, *P*. *jiugongshanensis*, *P*. *merrilliana*, *P*. *ranunculoides*) was closely related to Sects. *Crystallophlomis.* Li et al.^5^ conjectured that *P*. *hubeiensis* resembled *P*. *filchnerae*, and might belong in Sect. *Auganthus.* However, the present study clearly indicated that *P*. *hubeiensis* grouped with *P*. *ranunculoides* first, *P*. *cicutarrifolia* grouped with *P*. *merrilliana* first, and therefore *P*. *hubeiensis* should be placed in Sect. *Ranunculoides.* He et al*.*^4^ also demonstrated that *P*. *cicutarrifolia* was closely related with *P*. *merrilliana*. *P*. *ranunculoides* and *P*. *cicutarrifolia* were united into one species^2^ but later separated as two species^3^, and our ML and NJ analyses strongly supported the taxonomic treatment of Shao et al.^3^.

## Supplementary information

Supplementary Information.

## Data Availability

The complete chloroplast sequences generated and analyzed in this paper are available in GenBank (https://www.ncbi.nlm.nih.gov/genbank/, accession numbers listed in the text), the raw reads deposited in Genbank are SRR12179774–SRR12179778.
