# Supplementary material for: The complete chloroplast genome sequences of five pinnate-leaved Primula species and phylogenetic analyses
Source: Sci Rep. 2020 Nov 27;10:20782. doi: 10.1038/s41598-020-77661-3 (PMC7699626; doi:10.1038/s41598-020-77661-3)
Supplement: Supplementary file 1 — Supplementary Information. [file 41598_2020_77661_MOESM1_ESM.docx]

The complete chloroplast genome sequences of five pinnate-leaved *Primula* species and phylogenetic analyses

Wenbin Xu, Boshun Xia, Xinwei Li^*^

Wuhan Botanical Garden, Chinese Academy of Sciences, Wuhan 430074, China (^*^Corresponding author. E-mail address: forfortomorrow@163.com).

Abstract

The pinnate-leaved species (*P. cicutarrifolia*, *P*. *hubeiensis*, *P. jiugongshanensis*, *P. merrilliana*, *P*. *ranunculoides* and *P. filchnerae*) are a very particular group in the genus *Primula.* In the present paper, we sequenced, assembled and annotated the chloroplast genomes of five of them (*P. cicutarrifolia*, *P*. *hubeiensis*, *P. jiugongshanensis*, *P. merrilliana*, *P*. *ranunculoides*). The five chloroplast genomes ranged from ~ 150 to 152 kb, containing 113 genes (four ribosomal RNA genes, 29 tRNA genes and 80 protein-coding genes). The six pinnate-leaved species exhibited synteny of genes and possessed similar IR boundary regions in chloroplast genomes. The gene *accD* was pseudogenized in *P*. *filchnerae*. In the chloroplast genomes of the six pinnate-leaved *Primula* species, SSRs, repeating sequences and divergence hotspots were identified; *ycf1* and *trnH*-*psbA* were the most variable markers among CDSs and noncoding sequences, respectively. Phylogenetic analyses showed that the six *Primula* species were separated into two distant clades: one was formed by *P*. *filchnerae* and *P*. *sinensis* and the other clade was consisting of two subclades, one formed by *P*. *hubeiensis* and *P*. *ranunculoides*, the other by *P. merrilliana*, *P. cicutarrifolia* and *P*. *jiugongshanensis*. *P*. *hubeiensis* was closely related with *P*. *ranunculoides* and therefore it should be placed into Sect. *Ranunculoides*. *P. cicutarrifolia* did not group first with *P*. *ranunculoides* but with *P. merrilliana*, although the former two were once united in one species, our results supported the separation of *P*. *ranunculoides* from *P. cicutarrifolia* as one distinct species.

Key words: *Primula*, chloroplast genome, phylogeny, pinnate-leaved species.


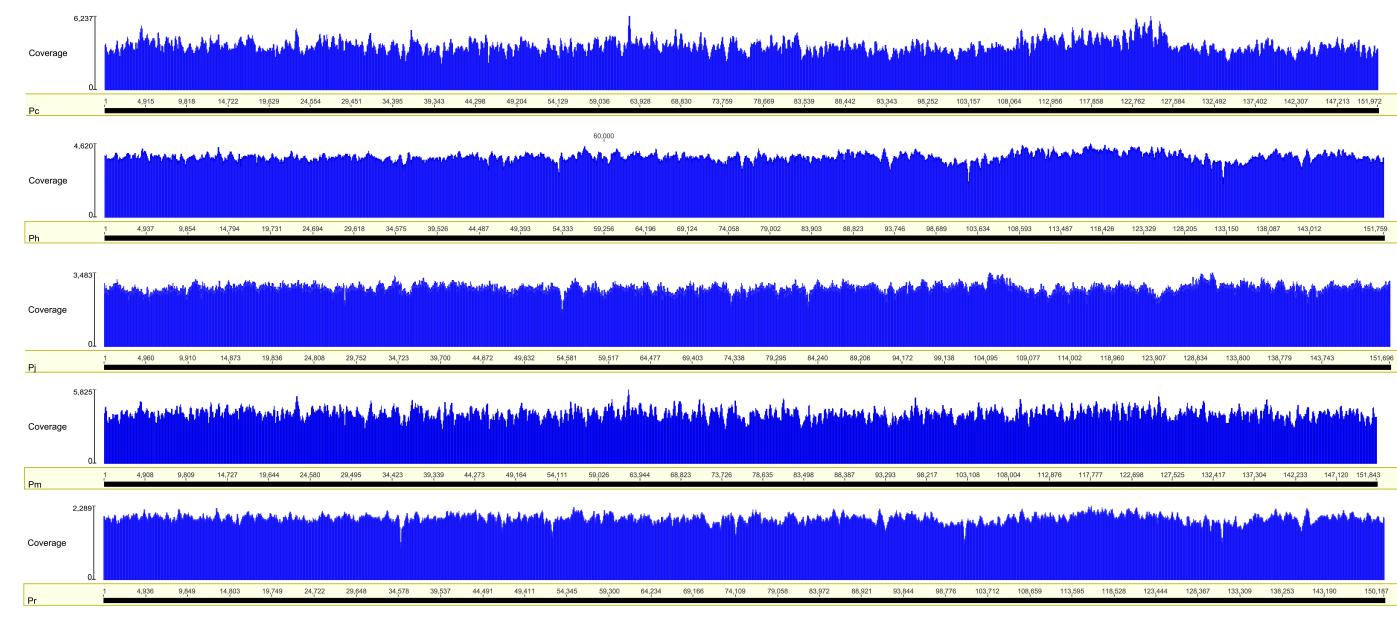


Figure S1 Sequencing coverage of the five cp genomes estimated by mapping filtered data to the five cp genomes in Geneious R9. Pc: *P*. *cicutarrifolia*; Ph: *P*. *hubeiensis*; Pj: *P*. *jiugongshanensis*; Pm: *P*. *merrilliana*; Pr: *P*. *ranunculoides*.


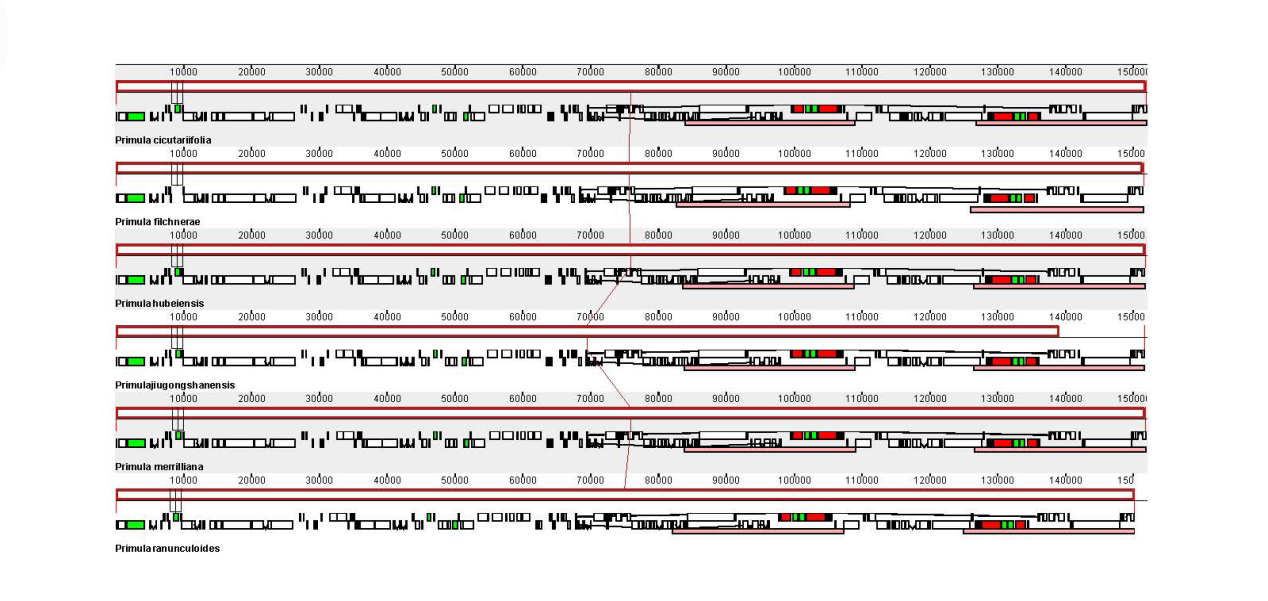


Figure S2 MAUVE alignment of six *Primula* species chloroplast genomes.


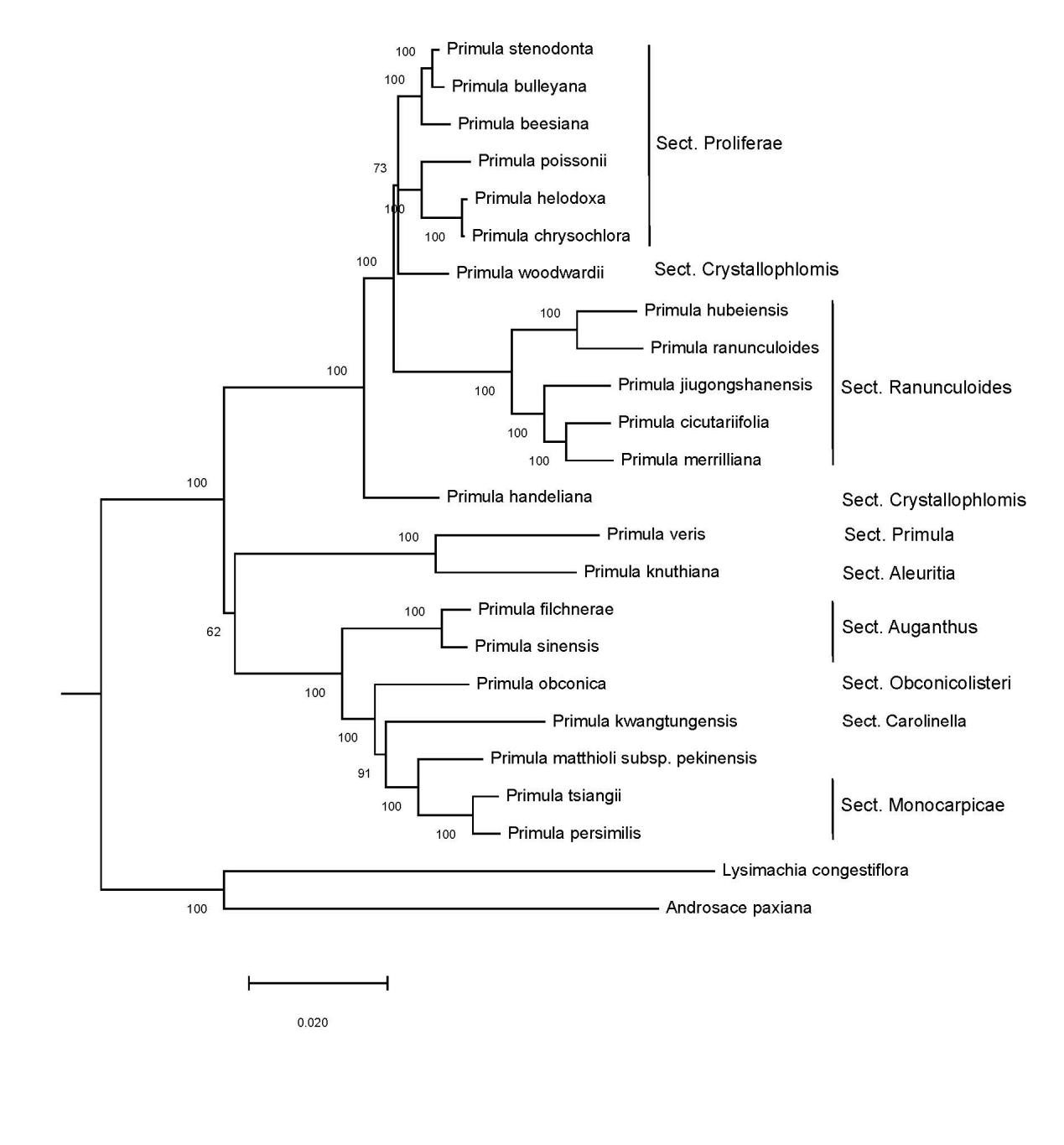


Figure S3 ML phylogenetic tree of *Primula* species based on the gene *ycf1*.

Table S1. NCBI accessions of chloroplast genomes for sequence divergence analysis and ML phylogenetic tree construction.

| Species | accession |
| --- | --- |
| *Androsace paxiana* | KU513437 |
| *Lysimachia congestiflora* | NC_045275 |
| *Primula beesiana* | MN504639 |
| *Primula chrysochlora* | NC_034678 |
| *Primula handeliana* | NC_039348 |
| *Primula helodoxa* | MN504640 |
| *Primula knuthiana* | NC_039350 |
| *Primula kwangtungensis* | NC_034371 |
| *Primula matthioli* subsp. *pekinensis* | KY235373 |
| *Primula obconica* | KX618681 |
| *Primula persimilis* | NC_034331 |
| *Primula poissonii* | NC_024543 |
| *Primula sinensis* | NC_030609 |
| *Primula stenodonta* | NC_034677 |
| *Primula tsiangii* | MN065496 |
| *Primula veris* | NC_031428 |
| *Primula woodwardii* | NC_039349 |
| *Primula bulleyana* | MN428416 |
| *Primula cicutariifolia* | MT268974 |
| *Primula filchnerae* | MK888698 |
| *Primula hubeiensis* | MT268976 |
| *Primula jiugongshanensis* | MT937162 |
| *Primula merrilliana* | MT268977 |
| *Primula ranunculoides* | MT268978 |

Table S2. NCBI accessions of gene fragments used for ML and NJ phylogenetic tree construction. Conceptions of sections and subgenera follow Hu & Kelso (1996) and Richards (2003), respectively.

| Species | Subgenus | Section | *rbcL* | *matK* | ITS |
| --- | --- | --- | --- | --- | --- |
| Primula fasciculata | Subgenus *Aleuritia* | Section *Aleuritia* | JF943647 | JF955710 | JF977991 |
| Primula gemmifera | Subgenus *Aleuritia* | Section *Aleuritia* | JF943651 | JF955714 | JF977994 |
| Primula kialensis | Subgenus *Aleuritia* | Section *Aleuritia* | JF943655 | JF955718 | JF977998 |
| Primula membranifolia | Subgenus *Aleuritia* | Section *Aleuritia* | JF943664 | JF955727 | JF978007 |
| Primula munroi | Subgenus *Aleuritia* | Section *Aleuritia* | JF943670 | JF955733 | JF978013 |
| Primula prattii | Subgenus *Aleuritia* | Section *Aleuritia* | JF943690 | JF955753 | JF978033 |
| Primula pulchella | Subgenus *Aleuritia* | Section *Aleuritia* | JF943695 | JF955758 | JF978038 |
| Primula yunnanensis | Subgenus *Aleuritia* | Section *Aleuritia* | JF943727 | JF955792 | JF978068 |
| Primula agleniana | Subgenus *Aleuritia* | Section *Crystallophlomis* | JF943586 | JF955649 | JF977932 |
| Primula boreio_calliantha | Subgenus *Aleuritia* | Section *Crystallophlomis* | JF943611 | JF955674 | JF977957 |
| Primula chionantha | Subgenus *Aleuritia* | Section *Crystallophlomis* | JF943625 | JF955688 | JF977971 |
| Primula diantha | Subgenus *Aleuritia* | Section *Crystallophlomis* | JF943637 | JF955700 | JF977983 |
| Primula melanops | Subgenus *Aleuritia* | Section *Crystallophlomis* | JF943662 | JF955725 | JF978005 |
| Primula szechuanica | Subgenus *Aleuritia* | Section *Crystallophlomis* | JF943720 | JF955785 | JF978063 |
| Primula calliantha | Subgenus Aleuritia | Section Crystallophlomis | JF943618 | JF955681 | JF977964 |
| Primula denticulata | Subgenus *Aleuritia* | Section *Denticulata* | JF943633 | JF955696 | JF977979 |
| Primula bella | Subgenus *Aleuritia* | Section *Minutissimae* | JF943599 | JF955662 | JF977945 |
| Primula bellidifolia | Subgenus *Aleuritia* | Section *Muscarioides* | JF943602 | JF955665 | JF977948 |
| Primula deflexa | Subgenus *Aleuritia* | Section *Muscarioides* | JF943629 | JF955692 | JF977975 |
| Primula calderiana | Subgenus *Aleuritia* | Section *Petiolares* | JF943615 | JF955678 | JF977961 |
| Primula epilosa | Subgenus *Aleuritia* | Section *Petiolares* | JF943641 | JF955704 | JF977985 |
| Primula moupinensis | Subgenus *Aleuritia* | Section *Petiolares* | JF943665 | JF955728 | JF978008 |
| Primula ovalifolia | Subgenus *Aleuritia* | Section *Petiolares* | JF943679 | JF955742 | JF978022 |
| Primula sonchifolia | Subgenus *Aleuritia* | Section *Petiolares* | JF943716 | JF955781 | JF978059 |
| Primula tardiflora | Subgenus *Aleuritia* | Section *Petiolares* | JF943722 | JF955787 | JF978065 |
| Primula anidosora | Subgenus *Aleuritia* | Section *Proliferae* | KP638648 | KP638608 | KP638568 |
| Primula aurantiaca | Subgenus *Aleuritia* | Section *Proliferae* | HM018322 | HM018224 | HM018175 |
| Primula beesiana | Subgenus *Aleuritia* | Section *Proliferae* | KP638651 | KP638611 | KP638571 |
| Primula bulleyana | Subgenus *Aleuritia* | Section *Proliferae* | KP638653 | KP638613 | KP638573 |
| Primula burmanica | Subgenus *Aleuritia* | Section *Proliferae* | KP638654 | KP638614 | KP638574 |
| Primula chrysochlora | Subgenus *Aleuritia* | Section *Proliferae* | KP638656 | KP638616 | KP638576 |
| Primula chungensis | Subgenus *Aleuritia* | Section *Proliferae* | HM018323 | HM018225 | HM018176 |
| Primula cockburniana | Subgenus *Aleuritia* | Section *Proliferae* | KP638661 | KP638621 | KP638581 |
| Primula helodoxa | Subgenus *Aleuritia* | Section *Proliferae* | HM018325 | HM018227 | HM018178 |
| Primula mallophylla | Subgenus *Aleuritia* | Section *Proliferae* | KP638664 | KP638624 | KP638584 |
| Primula melanodonta | Subgenus *Aleuritia* | Section *Proliferae* | KP638666 | KP638626 | KP638586 |
| Primula miyabeana | Subgenus *Aleuritia* | Section *Proliferae* | KP638669 | KP638629 | KP638589 |
| Primula poissonii | Subgenus *Aleuritia* | Section *Proliferae* | HM018338 | HM018240 | HM018191 |
| Primula prenantha | Subgenus *Aleuritia* | Section *Proliferae* | KP638674 | KP638634 | KP638594 |
| Primula pulverulenta | Subgenus *Aleuritia* | Section *Proliferae* | HM018317 | HM018219 | HM018170 |
| Primula secundiflora | Subgenus *Aleuritia* | Section *Proliferae* | HM018352 | HM018254 | HM018205 |
| Primula serratifolia | Subgenus *Aleuritia* | Section *Proliferae* | HM018319 | HM018221 | HM018172 |
| Primula smithiana | Subgenus *Aleuritia* | Section *Proliferae* | HM018318 | HM018220 | HM018171 |
| Primula wilsonii | Subgenus *Aleuritia* | Section *Proliferae* | KP638682 | KP638642 | KP638602 |
| Primula alpicola | Subgenus *Aleuritia* | Section *Sikkimemsis* | JF943591 | JF955654 | JF977937 |
| Primula sikkimensis | Subgenus *Aleuritia* | Section *Sikkimemsis* | JF943708 | JF955773 | JF978051 |
| Primula spicata | Subgenus *Aleuritia* | Section *Soldanelloides* | JF943718 | JF955783 | JF978061 |
| Primula blinii | Subgenus *Aleuritia* | Section *Souliei* | JF943609 | JF955672 | JF977955 |
| Primula filchnerae | Subgenus *Auganthus* | Section *Auganthus* | MK888698 | | MT298893 |
| Primula sinensis | Subgenus *Auganthus* | Section *Auganthus* | JF943711 | JF955776 | JF978054 |
| Primula heucherifolia | Subgenus *Auganthus* | Section *Cortusoides* | JF943653 | JF955716 | JF977996 |
| Primula polyneura | Subgenus *Auganthus* | Section *Cortusoides* | JF943688 | JF955751 | JF978031 |
| Primula septemloba | Subgenus *Auganthus* | Section *Cortusoides* | JF943703 | JF955768 | JF978046 |
| Primula aromatica | Subgenus *Auganthus* | Section *Malvacea* | JF943596 | JF955659 | JF977942 |
| Primula blattariformis | Subgenus *Auganthus* | Section *Malvacea* | JF943605 | JF955668 | JF977951 |
| Primula malvacea | Subgenus *Auganthus* | Section *Malvacea* | JF943660 | JF955723 | JF978003 |
| Primula duclouxii | Subgenus *Auganthus* | Section *Monocarpicae* | JF943639 | JF955702 | KM198444 |
| Primula obconica | Subgenus *Auganthus* | Section *Obconicolisteri* | JF943674 | JF955737 | JF978017 |
| Primula oreodoxa | Subgenus *Auganthus* | Section *Obconicolisteri* | JF943677 | JF955740 | JF978020 |
| Primula pycnoloba | Subgenus *Auganthus* | Section *Pycnoloba* | JF943698 | JF955761 | JF978041 |
| Primula amethystina | Subgenus *Auriculastrum* | Section *Amethystina* | JF943593 | JF955656 | JF977939 |
| Primula faberi | Subgenus *Auriculastrum* | Section *Amethystina* | JF943644 | JF955707 | JF977988 |
| Primula chapaensis | Subgenus *Carolinella* | Section *Carolinella* | JF943620 | JF955683 | JF977966 |
| Primula partschiana | Subgenus *Carolinella* | Section *Carolinella* | JF943684 | JF955747 | JF978027 |
| Primula rugosa | Subgenus *Carolinella* | Section *Carolinella* | JF943701 | JF955764 | JF978044 |
| Primula wangii | Subgenus *Carolinella* | Section *Carolinella* | JF943726 | JF955791 | JF978067 |
| Primula hubeiensis | Subgenus Pinnatae | Sect. Ranunculoides | MT268976 | | MT298894 |
| Primula ranunculoides | Subgenus Pinnatae | Sect. Ranunculoides | MT268978 | | MT298896 |
| Primula merrilliana | Subgenus Pinnatae | Sect. Ranunculoides | MT268977 | | MT298892 |
| Primula cicutariifolia | Subgenus Pinnatae | Sect. Ranunculoides | MT268974 | | MT298895 |
| Primula jiugongshanensis | Subgenus Pinnatae | Sect. Ranunculoides | MT937162 | | MT928878 |
| Androsace paxiana |  |  | KU513437 | | AF323705 |
| Lysimachia congestiflora |  |  | NC_045275 | | FJ529519 |
